# The dynamics of competition and decision-making

**DOI:** 10.3758/s13423-024-02523-2

**Published:** 2024-06-10

**Authors:** Andrew J. Morgan, Andrew Neal, Timothy Ballard

**Affiliations:** School of Psychology, University of Queensland, Brisbane, Australia

**Keywords:** Computational modeling, Decision making, Competition, Dynamics

## Abstract

We examine the underlying cognitive mechanisms that govern how competitions play out over time. We used cognitive modeling to examine the dynamic effects of time remaining and relative performance (whether the person is winning or losing) on effort and strategy. In this experiment, participants completed a competitive decision-making task with varying time limits and starting scores, in a repeated-measures design. Participants were tasked with scoring more points than their computerized opponent during a certain time frame, gaining and losing points for correct and incorrect decisions, respectively. The results showed that as the competition deadline approached and as participants drew ahead of their opponent within a competition, they increased effort and became more cautious. Furthermore, the effect of relative score on effort and caution changed over the course of a competition as the deadline approached. These results highlight the importance of considering dynamics when working to understand how competitions unfold as well as the underlying cognitive mechanisms that give rise to the dynamic behavior.

**Supplementary Information:**

The online version contains supplementary material available at 10.3758/s13423-024-02523-2.

Competition occurs when multiple individuals are working to gain resources that cannot be evenly distributed between them (Deutsch, 1949). Competition is inherently dynamic. Each competitor’s relative status changes over time as they work to gain an advantage and there are multiple ways competitors might respond to these changes. Consider a day trader competing with colleagues to earn the most profit in a day. As the day ends, a competitor whose earnings are lagging may need to change course to catch up. They may attempt to increase their rate of earnings by expending more effort to make better trades, or by adjusting their strategy by spending less time deliberating to maximize the number of trades they can execute. The dynamics by which competitors’ effort and strategies change have a profound impact on how competitions unfold, but they are not well understood.

The vast majority of past research has treated competition as static, focusing only on factors that predict motivational or performance outcomes. This research, for example, has contrasted co-operation and competition, finding co-operation to be better for task achievement and satisfaction (Haines & McKeachie, 1967; Johnson et al., 1981; Scott & Cherrington, 1974), while others found competition improved performance and motivation (Erev et al., 1990; Tauer & Harackiewicz, 2004). Yet very little work has examined the underlying dynamics that determine how competitions evolve over time. Previous work on competition emphasized the question of *what* the likely outcomes are but neglected the question of *how* those outcomes emerge.

This study examines the dynamics of competition in the context of decision-making, specifically how effort and strategy change over time in response to closing deadlines and relative performance between the competitor and their opponent. To achieve this, we ran an experiment in which participants made a series of decisions to earn points, with the goal being to have more points than a computerized opponent at the end of a fixed timeframe. We used cognitive modeling to quantify changes in effort and strategy over time as a function of relative status and time to deadline.

## The dynamics of competition

Competition is most often studied by examining its effect on motivation or, more commonly, performance, usually by comparing them in the presence of competition or co-operation. For example, Tauer and Harackiewicz (2004) examined the effects of competition, co-operation, and intergroup competition on enjoyment and performance in a basketball task. They found no differences between cooperation and competition on these two outcomes, though the combination of cooperation and competition (intergroup competition) lead to higher enjoyment of the task, and in some cases, higher performance. Another study by Robie et al. (2005) examined competitiveness and performance in the context of sales consultants. Using survey measures to assess competitiveness and interdependence (desire to work in co-operative group settings), as well as using preexisting sales data as performance, they found more competitive consultants tended to perform better. Other research has identified factors that moderate the effects of competition such as goals (Brown et al., 1998), self-confidence (Tuckman, 2003), and trait competitiveness (Kilduff, 2014).

Multiple meta-analyses have examined the impact of competition on performance. Johnson et al. (1981) showed that cooperation and group-based competition led to better achievement and productivity than individual competition or goal setting. However, a more recent meta-analysis found no direct impact of competition on performance, due to opposing mediation effects that cancel out (Murayama & Elliot, 2012). Competition induced a mindset of trying to appear competent towards others, improving performance, but also increased a mindset of avoiding appearing incompetent towards others, undermining performance. While this work acknowledges an underlying mechanism that drives performance during competition, the studies examined tend to use between-person designs with one-time measures of competition and performance that do not actually examine the dynamics of how this process plays out. This limits our ability to make conclusions about how competitions unfold.

A small number of studies have attempted to examine the dynamics of competition using designs that track motivation and performance over time. For example, Huang et al. (2017) assessed how an individual’s standing in a competition impacted their motivation through a multiphase dice game against an opponent, where winning meant having more points than their opponent after five rounds. They were given fake feedback that they were ahead or behind their opponent, by either 30 or 60 points, and either after an early or a later round. Motivation was measured by employing a bonus round where the longer the participant waited in the round, the more points they could earn; the longer wait times were said to be indicative of higher motivation. They found that being ahead in the competition early on increased motivation to win the task, but decreased motivation in the later stages. These effects were attributed to winning being perceived as more attainable and by reducing the perceived effort required to win, respectively.

The above study provides some evidence that one’s position relative to their opponent impacts motivation during a competition, and that this relationship may change over time. While this study did allow for some examination of competition dynamics by manipulating feedback timing and relative position, it did not allow for examination of the continuous change over the course of a competition. It used a between-participants design which did not examine how these processes examine change over time within a person. Furthermore, studies that use a broad measure of ‘motivation’ as their outcome variable fail to differentiate between the different motivational dynamics at play.

Motivation refers to the direction, intensity, and persistence of behavior (Humphreys & Revelle, 1984). Direction determines the task that receives priority at a given point in time. For cognitive tasks, intensity reflects the amount of attention, concentration, or mental resources a person expends on a task. Persistence reflects the amount of time the person is willing to invest those resources in the task. Accordingly, there are multiple ways that changes in motivation might influence behavior over time during a competition. For example, in response to a looming deadline or an increasingly unfavorable position, people might become less persistent in terms of the time they spend gathering information before making a decision, opting for a quicker decision-making strategy by reducing caution. This approach would align with how people respond when motivated to avoid a punishment (Ballard et al., 2019). Alternatively, they might persist more, becoming more cautious, taking time to make more careful decisions to avoid errors, as has been observed when people are motivated by reward. People might also respond by increasing their intensity. For example, the person might expend more effort, allocating more cognitive resources and attempting to process information more rapidly in response to time pressure (Dambacher & Hübner, 2015). When outperforming their opponent, they might reduce intensity and expend less effort due to complacency (Berger et al., 2013).

### The current study

Unpacking the above dynamics requires a within-person design that tracks effort and strategy over time across a series of decisions that people make during a competition. In the current study, we do just this. Participants engaged in a competitive decision-making task against a computerized opponent where they could gain or lose points for correct or incorrect decisions, respectively. Their objective was to accumulate the most points by the end of an explicit time limit. We varied the time limit for the competitive episodes, as well as the starting scores for the participant and the opponent.

**Fig. 1:**
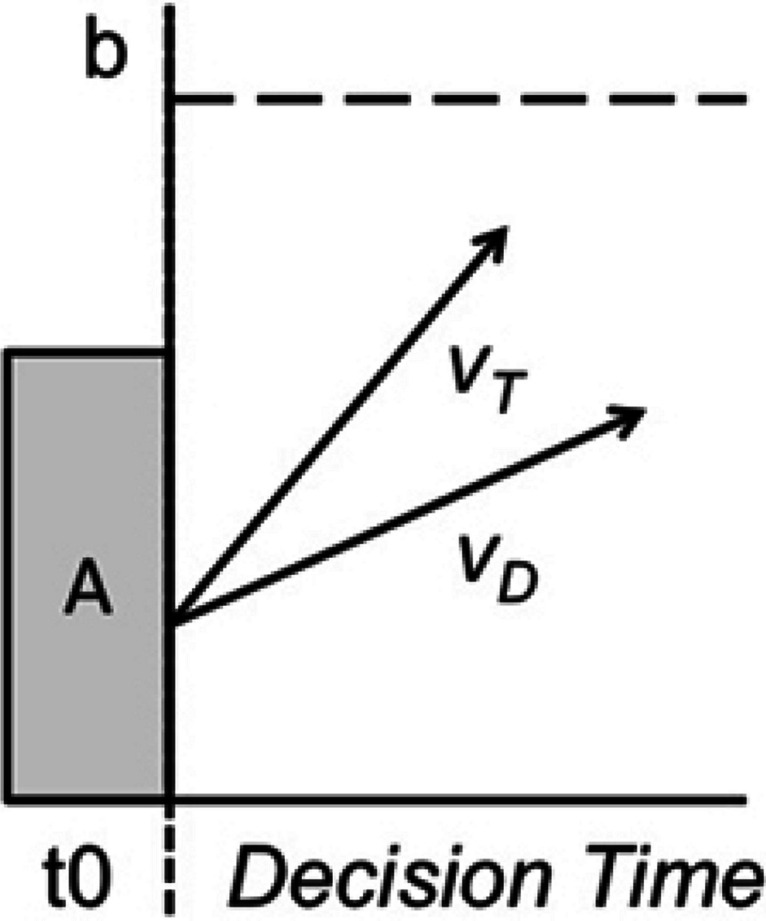
The linear ballistic accumulator model. The b parameter represents the threshold for decision-making, and the V parameters represent the rate of evidence accumulation. Once enough evidence is accumulated (when V crosses b), a decision is made. The A parameter represents the randomized starting point of evidence for a decision (V can start at any point within A’s boundaries), and t0 represents nondecision time, when participants are first perceiving the stimulus

We used cognitive modeling to quantify the relationships between relative score and time remaining on effort and strategy. This involved modeling of decision thresholds and rates of evidence accumulation using the Linear Ballistic Accumulator (LBA; see Fig. 1). Evidence accumulation models provide frameworks to disentangle the effects of strategy and effort during decision-making (Brown & Heathcote, 2008; Ratcliff & McKoon, 2008; Usher & McClelland, 2001). They assume decisions are made by sampling information over time until enough evidence is accumulated to reach a decision-making threshold, at which point a decision is made. The LBA (Brown & Heathcote, 2008) assumes evidence accumulates linearly and separately for each individual response. The rate at which this evidence accumulates indexes the speed of information processing, providing a measure of the effort one invests into a task (Ballard et al., 2019; Eidels et al., 2010; Palada et al., 2016). An increase in the rate of evidence accumulation produces an increase in response speed, while an increase in the difference between the drift rates for the correct and incorrect responses produces an increase in accuracy, as evidence for the correct response will tend to accumulate more quickly. The threshold for making a response provides an index of strategy. Higher thresholds reflect a more cautious strategy. Increasing threshold means more evidence must be accumulated before a decision is made. This produces slower and more accurate decisions. Lower thresholds reflect a less cautious strategy, producing faster and less accurate decisions.

## Method

### Participants

This sample consisted of 70 participants who were undergraduate psychology students at an Australian university and who participated for course credit. Four participants’ data were removed from the analyses as their performance was not meaningfully above chance.^1^ This resulted in 66 participants (14 male and 52 female, mean age of 19.7 years). The preregistration for this study can be found online (https://osf.io/7tj9z).

### Experimental task

The experiment used the random dot task, a perceptual decision-making task that requires participants to make quick and accurate decisions in response to stimuli presented on a computer screen (Holmes et al., 2016). Participants were presented with a cloud of rapidly moving dots and indicated whether they were moving to the right or left. Ten percent of the dots were moving in the same direction with the remaining dots moving in random directions. The stimuli we used consisted of 200 dots that were each four pixels wide in an aperture that is 1,000 pixels wide and 500 pixels high. Each dot moved three pixels per frame and moved along its trajectory (randomly or coherently left/right) for 20 frames before being reset at a random location. The original code for the task was adapted from Rajananda et al. (2018).^2^

These decisions were made using a key press input on a keyboard. The participants pressed the “A” key if they believed the coherent dots were moving left and the “L” key if they believed the coherent dots were moving right. Each correct decision earned the participant a point while each incorrect decision lost them a point. They also received audio feedback (a “ding” sound for correct decisions and a “buzz” sound for incorrect decisions).

Participants competed against a computerized opponent. We used a computer opponent to ensure consistency across participants in how difficult it was to win the competition. Computerized opponents have been used in previous research using similar tasks (Morgan et al., 2023). Research suggests that humans often interact with computers in ways that are similar to how they interact with other humans (Nass et al., 1994; Posard & Rinderknecht, 2015), tending to anthropomorphize the computerized agent and treating it as a social actor (Cogoni et al., 2024). We return to potential limitations of computerized opponents in the Discussion.

The computer opponent’s decisions were generated using the LBA with parameter values that were determined by fitting the model to data from a pilot study (see Supplementary Materials). The opponent therefore produced a pattern of choices and response times that was typical of a human participant in this task. In each competitive round, the participant’s goal was to have a score greater than their opponent at the end of the allotted time. The computer opponent performed at a level that was based on pilot testing participant data.^3^ On average, the opponent reached a score of eight points in 20 seconds.

**Fig. 2:**
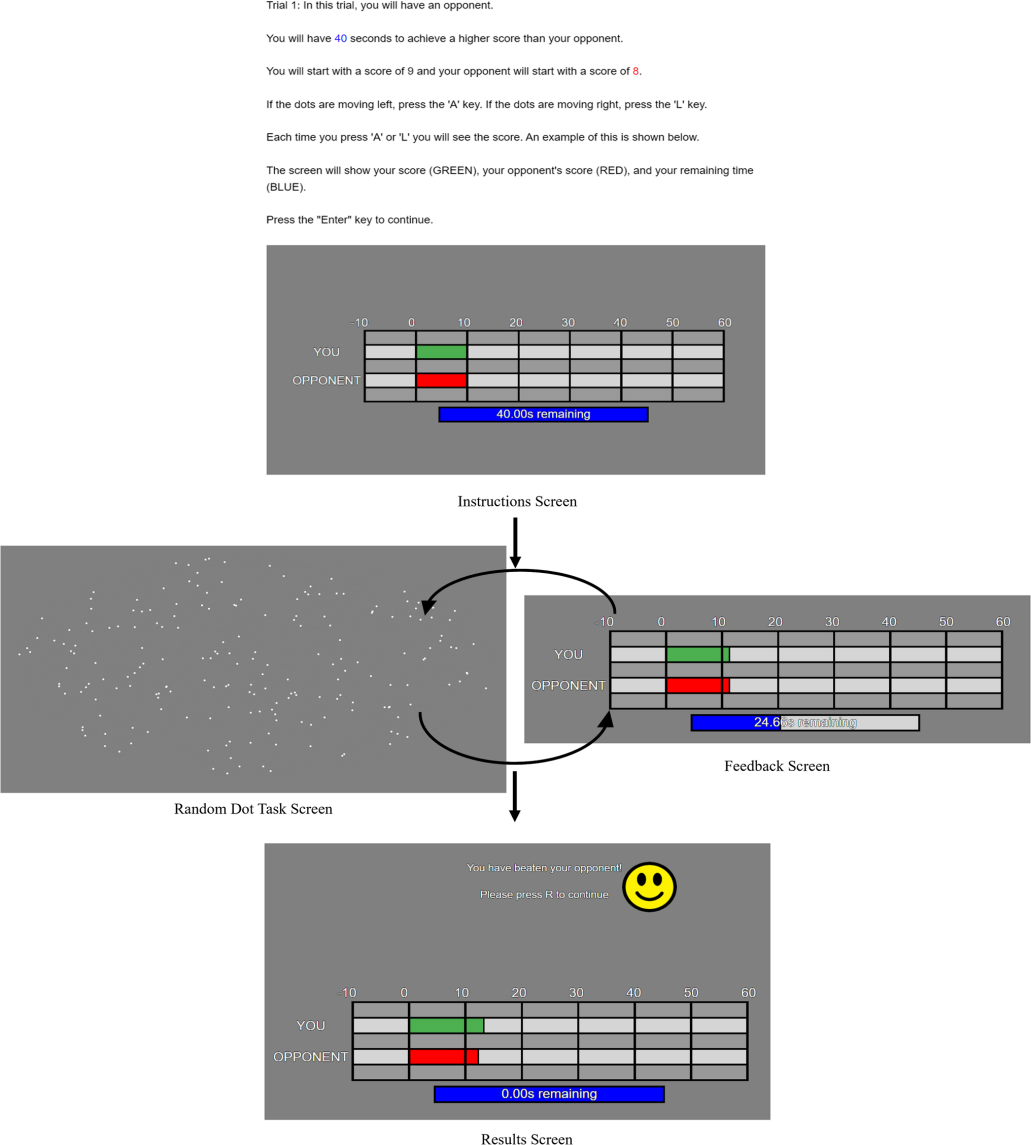
Task screens for the experiment. Participants are shown the instructions screen and are then taken to the random dot task screen, being shown the feedback screen after each response. At the end of the time limit, they are shown the results screen, and then move on to the next episode, starting at the instructions screen

At the start of the experiment, the participant had no knowledge regarding the ability of the computer opponent, but the participant could observe the opponent’s performance as the competitions played out. After each decision, the participant was shown a progress screen (presented for one second) indicating their score, their opponent’s score, and the time remaining. Once the time limit had run out, the participants were shown the results screen informing them of the final scores and whether they won, lost, or tied. They also received a prompt to press the “R” key to begin the next competition. The sequence of the experiment is shown in Fig. 2.

We manipulated the deadline across four levels: 5 seconds, 10 seconds, 20 seconds, and 40 seconds. We also manipulated the difference between the starting scores of the participant and their opponent. The starting difference ranged from negative six to positive six, with positive differences indicating that the participant had a higher score and negative difference indicating the opponent had a higher score. The within-person factorial manipulation of deadline (four levels) and starting score (13 levels) resulted in a total of 52 competitive episodes. The manipulations were included to make the task more engaging by creating variability in starting position and competition length.

### Procedure

Participants completed the task online, after signing up to participate through an internal university recruitment page for research participation. Participants read through an information sheet detailing what was required of them in the experiment. They then started the experimental task, received general instructions, and were asked to provide demographic information. A practice competition round was then completed before the 52 experimental episodes. Before each round, participants were told the deadline, their starting score, and their opponent’s starting score (the participant’s starting score was randomly generated between 7 and 13, and the opponent’s starting score was then calculated from this using the varying starting score difference). The order of the experimental episodes was randomized. After completing all the episodes, participants were debriefed about the task and given details on how to find out more information about the study.

The sample size was 56,414 total decisions across 3,432 experimental episodes and 66 participants (an average of 854.76 decisions per participant), which meets the recommended sample size for accurate estimation of parameters through the LBA (Donkin et al., 2009). In line with our preregistered exclusion criteria, practice episodes were excluded from the analyses, as were decisions that were too long (5 seconds or longer) or decisions that were too quick (250 ms or fewer). Responses that are very slow are indicative of attentional lapses, while times that are very quick suggest that the participant did not make a decision in response to the stimulus. Once these exclusion criteria were applied, 1.75% of the decisions were removed (after excluding participants with less than 55% accuracy and practice episodes). The experiment and analysis code, as well as the data itself, are all available publicly on the Open Science Framework (https://osf.io/7tj9z).

This project met the ethical requirements for conducting human research and legal requirements in Australia. It was approved by The University of Queensland Human Research Ethics Committee B, approval number 2018002018.

### Model

The parameter estimation for the LBA model was conducted in Stan (Annis et al., 2017; Carpenter et al., 2017) using hierarchical Bayesian methodology, with parameters being estimated separately for each individual but being drawn from common population distributions. The LBA assumes that evidence for either response alternative accumulates in separate accumulators independently of the other. In this experiment, there were two possible responses, left or right, giving two evidence accumulators. The starting evidence for either alternative response for each decision trial is taken from a uniform distribution [0, *A*]. From this starting point, the evidence accumulates linearly. Each accumulator has a rate of evidence accumulation (the drift rate), which is drawn from a normal distribution with mean *v* and standard deviation *sd*. Evidence is accumulated until enough evidence for one response breaches the threshold for a decision to be made, at which point, the response is made. In line with common practice (e.g., Brown & Heathcote, 2008), we express threshold *(B*; indexing strategy*)* as the difference between the raw threshold *(b)* and the maximum starting evidence (*A*), where *B* = *b − A*. This isolates the difference between the starting point and the raw threshold, which both may vary across individuals. In addition, the LBA also includes a parameter for nondecision time (*t*_*0*_), which captures the portion of response time that is attributed to other processes besides the decision-making process. These include encoding the stimulus and executing the response manually.

In this experiment, a version of the LBA was used where the mean rates of evidence accumulation for the correct decision (indexing effort) varied across decisions as a function of the time remaining currently in the block and the current score difference. Our analyses examined the effects that emerge as the competition unfolded over time. The LBA assumed that threshold (*B*) varied as a function of these two variables. The starting point variability (*A*), nondecision time (*t*_*0*_), and the mean rate of evidence accumulation for incorrect responses were constrained across all episodes to be equal. The standard deviation of the drift rate (*sv*) was fixed to one for all episodes and accumulators.

The parameters were estimated using a hierarchical Bayesian framework that assumed that parameters varied across individuals and were drawn from shared population distributions (see below). These population distributions for each parameter have two hyperparameters—location (μ) and scale (σ). The hyperparameters were chosen to be weakly informative. The parameters at the participant level were modeled using either normal or truncated normal distributions. Both the *A* and *B* parameters were set with a lower bound of 0 and no upper bound. The *t*_*0*_ parameter was constrained between 0.1 and 1, while the *v* parameter was not truncated at all. The priors used were based on methods used by Gronau, Heathcote, and Matzke (2019), and are presented in Appendix Table 2.

The regression equations for the threshold and drift rate analyses, where i represents participant and j represents decision, were:

$$\begin{array}{l}{Threshold}_{ij}= {\beta }_{0i}+ {\beta }_{1i}\left({score\;difference}_{ij}\right)+ {\beta }_{2i}\left({deadline}_{ij}\right)+ {\beta }_{3i}\left({score\;difference}_{ij}\times {deadline}_{ij}\right),\\ {Drift\;Rate\;(correct)}_{ij}= {\beta }_{4i}+ {\beta }_{5i}\left({score\;difference}_{ij}\right)+ {\beta }_{6i}\left({deadline}_{ij}\right)+ {\beta }_{7i}\left({score\;difference}_{ij}\times {deadline}_{ij}\right),\end{array}$$

where ${score\;difference}_{ij}$ and ${deadline}_{ij}$ represent the difference in scores (participant score minus opponent score) and the amount of time remaining in the episode at the time the stimulus was presented and the decision trial begins. Thus, ${score\;difference}_{ij}$ and ${deadline}_{ij}$ vary across decisions within a competitive episode, but do not vary within individual decision trials.

In total, there were 11 parameters estimated for each individual: starting point variability (*A*), nondecision time (*t*_*0*_), the mean drift rate for the incorrect response, the four regression parameters that determine threshold for a given decision (${\beta }_{0}$, ${\beta }_{1}$*, *${\beta }_{2}$*,* and ${\beta }_{3}$), and the four regression parameters that determine the mean drift rate for the correct response for that decision (${\beta }_{4}$, ${\beta }_{5}$*, *${\beta }_{6}$*,* and ${\beta }_{7}$).

The Savage–Dickey density ratio (Verdinelli & Wasserman, 1995) was used to compute Bayes factors (BF) for the LBA parameters and the regression coefficients. Lee and Wagenmakers’ (2013) classifications were used to describe the evidence strength. The 95% credible interval (CI) is also reported for each effect to evaluate magnitude.

## Results

### Accuracy and response time

**Fig. 3:**
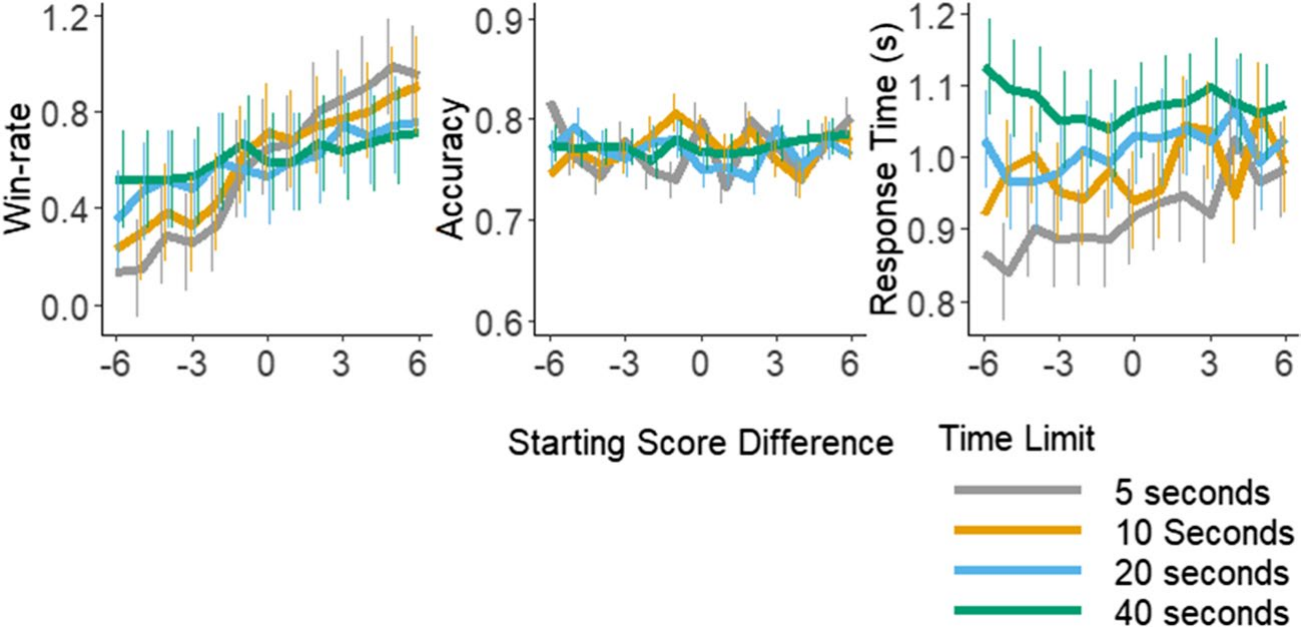
Average win rate, accuracy, and response time (in ms) across starting time limits and starting score differences. Win rates appear to have varied more when there was a shorter time limit, as did accuracy, while response times appear to have been longer when there was a longer overall time limit. (Color figure online)

The average accuracy across all decisions was 77.1%, with a standard deviation of 0.4%, and the average response time was 1.03 seconds, with a standard deviation of 0.65. The average participant win rate was 59.4%, with a standard deviation of 27.2%. Across the starting time limits, average win rates ranged from 58.0% (5 seconds) to 60.7% (40 seconds). Across the starting score differences, average win rates ranged from 30.7% (six points behind the opponent) to 83.3% (six points ahead of the opponent). The mean win rates, accuracies, and response times across the starting time limits and starting score differences are shown in Fig. 3.

**Fig. 4:**
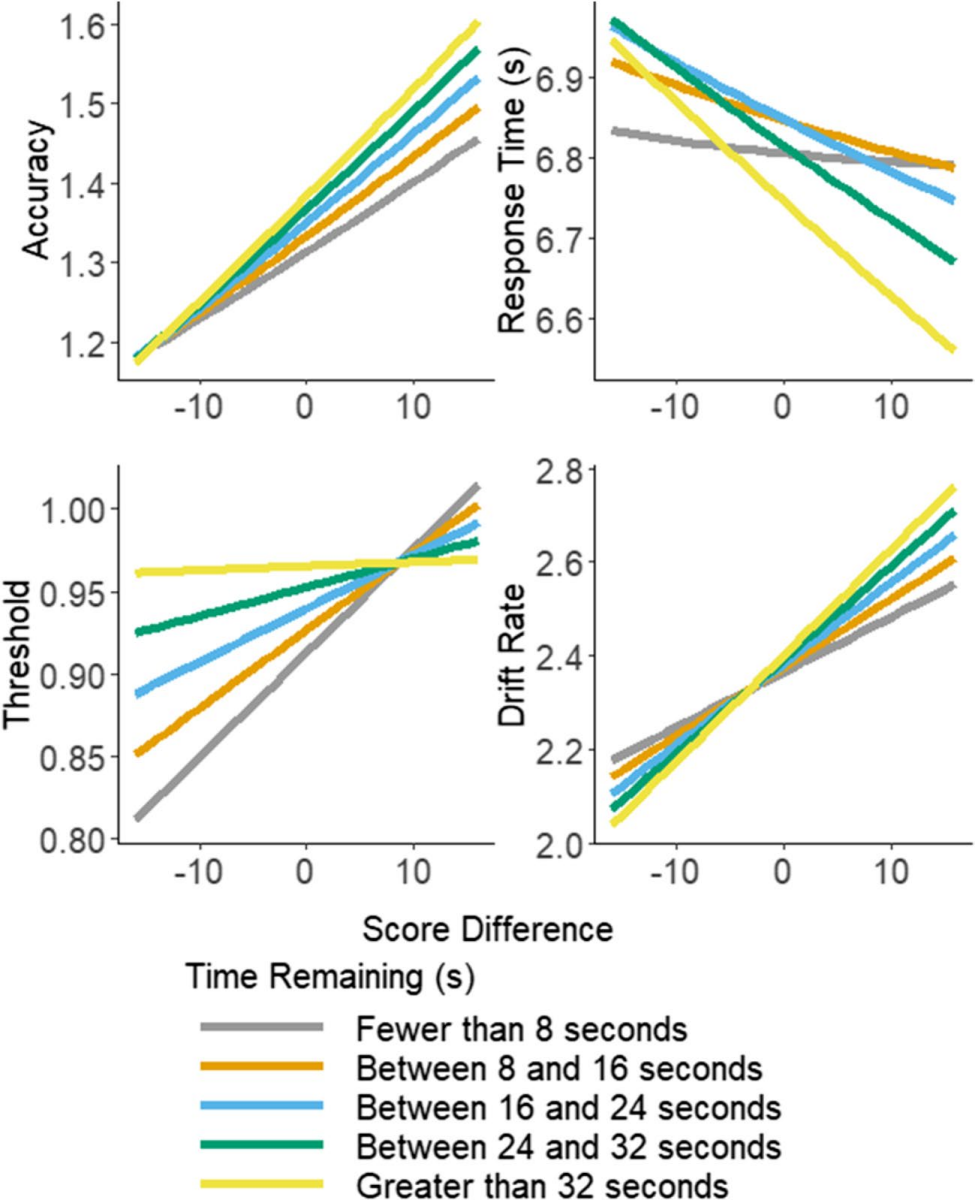
Line graphs of the effects of time remaining and score difference on accuracy, response time, threshold, and drift rate. The top left graph shows accuracy, the top right shows response time, the bottom left shows threshold, and the bottom right shows drift rate. (Color figure online)

To analyze the accuracy and response time effects, we used the brms package (Bürkner, 2017) in R (R Core Team, 2019) to run a pair of Bayesian mixed-effects polynomial regression (Edwards & Parry, 1993) models with time remaining in the block at the start of the decision trial, the current score difference between the participant and opponent, their interaction, and the squared terms of time remaining and score difference as predictors, with the participant identifiers entered as random effects (see Supplementary Material for information about priors). These models are separate from the LBA, regressing response time and accuracy directly on the predictor variables. The model of the accuracy data was run with a logit link function. There was very strong evidence for a positive effect of score difference, indicating that participants were more accurate when their score was higher than their opponent’s (see Table 1 and Fig. 4). There was very strong evidence for no effect of time remaining, no quadratic effects, and no interaction.

**Table 1:** Effects of time remaining, score difference, and their interaction on accuracy and response time. The null hypotheses for each coefficient (which were supported with a Bayes Factor below one) assume that the coefficients are equal to zero, while the alternative hypotheses for each coefficient (which were supported with a Bayes Factor above one) assume that the coefficients are not equal to zero

DV	Effect	Estimate	*SE*	Bayes factor	95% credible interval	Rhat
Accuracy	Intercept	1.24	0.06	525.85	1.13, 1.35	1.01
	Score difference	0.09	0.01	120.01	0.06, 0.12	1.00
	Score distance squared	0.002	0.005	0.0005	−0.008, 0.011	1.00
	Time remaining	0.009	0.01	0.001	−0.02, 0.03	1.00
	Time remaining squared	−0.0009	0.01	0.001	−0.02, 0.02	1.00
	Interaction	0.02	0.01	0.002	−0.01, 0.04	1.00
Response Time	Intercept	6.90	0.03	23.56	6.83, 6.97	1.13
	Score difference	−0.05	0.002	21.69	−0.055, −0.045	1.00
	Score distance squared	0.002	0.0008	0.002	0.0005, 0.0037	1.00
	Time remaining	0.05	0.002	15.93	0.04, 0.05	1.00
	Time remaining squared	−0.03	0.002	19.19	−0.04, −0.03	1.00
	Interaction	−0.03	0.002	23.56	−0.04, −0.03	1.00

*SE* represents the standard error. Lower CI and Upper CI represent the lower and upper bounds on the 95% credible interval. Rhat represents the R-hat convergence diagnostic, which is a comparison of the between- and within-chain estimate for the model parameters. BF represents the Bayes factor. Bayes factors were calculated using the Savage–Dickey density ratio method (Verdinelli & Wasserman, 1995)

Effects of time remaining, score difference, and their interaction on accuracy and response time The null hypotheses for each coefficient (which were supported with a Bayes factor below one) assume that the coefficients are equal to zero, while the alternative hypotheses for each coefficient (which were supported with a Bayes factor above 1) assume that the coefficients are not equal to zero.

The response time data was analyzed using the same model with a log link function. There was strong evidence for a negative effect of score difference, and for a positive effect of time remaining (see Table 1 and Fig. 4). Participants responded more quickly when their score was higher than their opponent’s and when there was less time remaining, on average. There was also strong evidence for an interaction between score difference and time remaining, whereby the effect of score difference weakens as time remaining decreases.

### Threshold and drift rate

Using the LBA, we found extreme evidence of effect of score difference at the time of the decision whereby participants set higher thresholds when they had greater time remaining, ${\beta }_{1i}$, BF = 4976, CI [0.03, 0.06], and higher thresholds when they had a higher score relative to their opponent, ${\beta }_{2i}$, BF = 2181.52, CI [0.03, 0.05], shown in Fig. 4. We found moderate evidence for an interaction between score difference and time remaining on threshold with the effect of score difference being stronger when less time remained, ${\beta }_{3i}$, BF = 3.44, CI [−0.03, −0.01].

We found extreme evidence of an effect of score difference at the time of the decision on drift rate for the correct response, where having a higher score relative to the opponent was associated with higher rates, ${\beta }_{5i}$, BF = 3861.49, CI [0.11, 0.16]. We also found strong evidence of an overall effect of time remaining at the time of the decision, where greater time remaining was associated with lower rates, ${\beta }_{6i}$, BF = 11.91, CI [−0.04, −0.01], though our parameter recovery analysis (see Supplementary Materials for full details) suggested that estimation of this parameter was somewhat less reliable than the others, meaning this result should be interpreted with caution, and be treated as non-diagnostic. Finally, we found moderate evidence of an interaction between the two variables on rate with the effect of score difference being weaker when less time remained, ${\beta }_{7i}$, BF = 6.92, CI [0.01, 0.06].

## Discussion

This research aimed to investigate the dynamics of effort and strategy over the course of a competition, by examining how they change over time as a function of closing deadlines and relative performance. To achieve this, we quantified effort and strategy using the LBA, which was fit to experiment data where people engaged in a competitive decision-making task. Justifying our distinction between different motivational dynamics, our results suggested both effort and strategy changed during the competition in different ways. Strategy changed in response to both relative scores and the deadline. When there was more time remaining in the competition, participants responded more cautiously by setting higher thresholds. As they had more time before the competition ended, they were able to take the time needed to maximize the chances of gaining each point. As time remaining wound down, participant’s thresholds decreased, and they made quicker, less cautious decisions in an attempt to secure more points. These results are consistent with findings suggesting more salient deadlines decrease response caution (Dambacher & Hübner, 2015).

Effort (operationalized as the drift rate for the correct response) also changed in response to both relative scores and the deadline. People expended more effort when they had higher scores relative to their opponents, consistent with previous work showing that people increased effort when performing better (Dissanayake et al., 2018; Huang et al., 2017), though this result goes against the notion that individuals get complacent when performing too well (Berger et al., 2013). This increase in effort could reflect participants trying harder in an attempt to secure their lead, or it could be a contrast to participants decreasing effort and giving up when they are performing worse. Our results also suggested that people may increase effort as deadlines approach, and allocate more cognitive resources to the task at hand, potentially reflecting an increased drive to secure points in response to the approaching end of the episode. As this parameter was estimated less reliably, further research is required to determine the robustness of this effect. In contrast to the strategy results, however, the relationship between relative score and effort weakened as the time remaining decreased. When the episode was almost over, winning participants were expending less effort than when there was more time remaining, potentially since there was a lower chance of losing their winning position. This suggests that people respond to discrepancies between their own position and their opponent’s by adjusting effort expenditure when under less time pressure, but by adjusting strategy when time pressure is greater. This finding is consistent with previous work showing that strategy is more likely adjusted when compared with effort when under a high degree of time pressure (Palada et al., 2018).

It is important to highlight that participants competed against a computer opponent and were told so before the competitions. We used this approach to achieve experimental control over the difficulty in beating the opponent. Prior research has shown similarities in how humans interact with computers versus other humans (Cogoni et al., 2024; Nass et al., 1994), and achieve similar self-reported positive affect, negative affect, and pleasantness (Kätsyri et al., 2013), when competing against a computer opponent compared with a human opponent. However, other work suggests that aggression (Williams & Clippinger, 2002), feelings of flow and enjoyment (Weibel et al., 2008), and neural responses to winning (Kätsyri et al., 2013), may differ when competing against computers compared with other humans. It may be that some participants are less invested when facing a computerized opponent than they would be when facing a human, having potential implications for the generalizability of our findings. Additionally, the study did not measure overall motivation nor induce motivation via incentives, so the influence of overall motivation’s influence on these effects remains to be determined. Finally, while we examined the dynamics of effort and strategy across decisions, there might also be shorter-term effects arising within decisions. Future research may investigate if and how effort and strategy change as evidence accumulates, and the effects these have on the competition’s dynamics.

Competition has long been treated as static in research, and the dynamics of how competitions play out over time have often been ignored. In addition, the underlying cognitive processes that guide decision-making in completive settings have also been overlooked. The current research is a first step towards understanding the combined role of decision-making and dynamics in competition. We have built on previous work examining the “what” of competition by exploring the “how” (i.e., the dynamics) of competition. A natural continuation of this line of work is investigation of the “who” by exploring whether the opponents themselves matter. For example, these motivational dynamics may play out differently when competing against a rival compared with a friend. Previous research has found that collaborating with friends versus nonfriends improved collaboration, so social relationships could impact the competitive side too (Brennan & Enns, 2015). In addition, the “why,” or the motivation and goals for competing, could also provide further insight using our framework. The types of goals individuals set, in terms of appearing competent or avoiding appearing incompetent likely impact behavior during competition, and these goals themselves could also change dynamically over time.

## Supplementary Information

Below is the link to the electronic supplementary material.

Supplementary file1 (DOCX 281 KB)

## Data Availability

All the data for this experiment is publicly available on the Open Science Framework (https://osf.io/7tj9z).

## Appendices

### Appendix 1: Model priors

**Table 2:** Priors for the population distributions

Population distribution	Model parameter	Distribution family	Mean	*SD*	Lower	Upper
Location	*A*	Truncated Normal	1	1	0	None
	*B intercept*	Truncated Normal	0.4	0.4	0	None
	*B on deadline*	Standard Normal	0	1	None	None
	*B on score difference*	Standard Normal	0	1	None	None
	*B on interaction*	Standard Normal	0	1	None	None
	*v true intercept*	Normal	3	3	0	None
	*v true on deadline*	Standard Normal	0	1	None	None
	*v true on score difference*	Standard Normal	0	1	None	None
	*v true on interaction*	Standard Normal	0	1	None	None
	*v false*	Normal	1	1	None	None
	*t*_*0*_	Truncated Normal	0.3	0.3	0.1	1
Scale	*A*	Truncated Normal	1	1	0	None
	*B*	Truncated Normal	0.4	0.4	0	None
	*v true*	Truncated Normal	3	3	0	None
	*v false*	Truncated Normal	1	1	0	None
	*t*_*0*_	Truncated Normal	0.3	0.3	0	None

The posterior distributions were estimated using the Hamiltonian Monte Carlo algorithm implemented by Stan (Annis et al., 2017; Carpenter et al., 2017). Four chains were run, each with 2,000 iterations; 1,000 of these iterations were run for sample burn in, and were discarded, leaving 1,000 iterations. Visual inspection of the chains showed excellent mixing and stationarity

## Footnotes

1 These data were removed due to being close to chance performance. Although this was not a part of our preregistration, the model failed to converge when these participants were included. Removal of low accuracy data is in line with previous studies (Bhui, 2019; Musslick et al., 2019; Trueblood et al., 2021). The removed participants’ overall accuracies were 44.68%, 47.88%, 48.00%, and 50.88%. The next lowest average accuracy was 58.41%.

2 While we have used Rajananda et al.’s (2018) random dot stimulus task, many previous studies are based on Newsome and Pare’s (1988) random dot stimulus task.

3 The computer opponent was determined by fitting the LBA to pilot data from real participants and using the obtained parameters to simulate the opponent decision-making. This makes the opponent performance on par with an average participant. See Supplementary Materials for more details.
