# Supplementary material for: The dynamics of competition and decision-making
Source: Psychon Bull Rev. 2024 Jun 10;31(6):2811–22. doi: 10.3758/s13423-024-02523-2 (PMC11680669; doi:10.3758/s13423-024-02523-2)

**Pilot Study**

In order to develop the stimuli for the experiment, we ran four pilot studies to determine an appropriate difficulty for the random dot discrimination and the computerized opponents. This process was done for a previous study, and the outcome variables have been carried over to this study. The first pilot test included three trials of ten different proportions of dots moving coherently in the Random Dot Motion Task. The coherences ranged from .1 to 1 in incremental steps of .1. Participants had 20 seconds to achieve a goal of 10 points and were not competing with an opponent. After having 22 undergraduate participants complete the pilot, we observed the data to find a coherence that led to approximately 70% accuracy and goal achievement 50% of the time. Accuracy and response times are shown below in Figure 1.

**Figure 1**

*Response time and accuracy data from the first pilot study.*
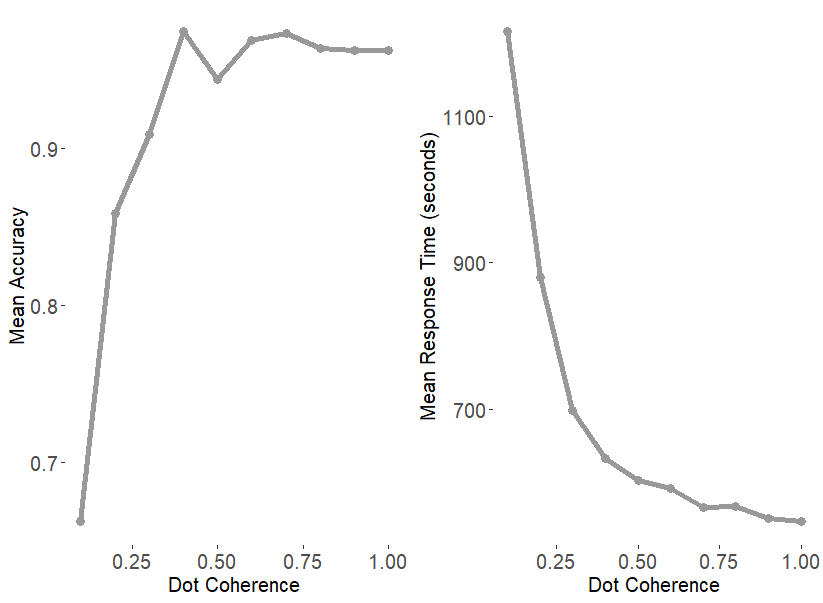


We concluded that the desired difficulty occurred somewhere below .2 and proceeded to pilot test the coherences from .04 to .26 in steps of .01. Each coherence had two episodes and maintained the time limit and goal from the first pilot. This second pilot had a sample of 23 undergraduate participants, and the accuracy and response times are shown below in Figure 2.

**Figure 2**

*Response time and accuracy data from the second pilot study.*
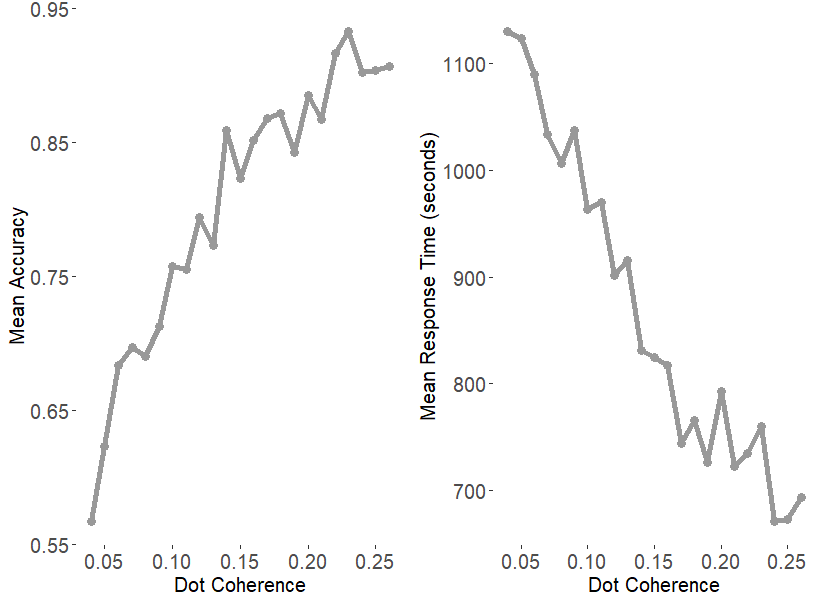


The .075 coherence trials appeared to have the required difficulty and was used for the next pilot. We had 12 undergraduate participants complete 30 trials at .075 coherence. The time limit remained at 20 seconds, though the goal was decreased to eight points, as approximately 54.7% of trials with coherences of .07 and .08 ended with at least eight points, roughly meeting our desired 50% goal achievement criteria. The purpose of this third pilot was to determine an appropriate difficulty for the computer opponent, based on a model of how the average participant would perform on the task. Upon analyzing the data, however, it appeared this coherence was more difficult than anticipated, with only 19.4% of trials ending in the participant achieving the set goal. This prompted us to lower the difficulty by raising the coherence to .1, and we ran another pilot study. We had ten undergraduate participants complete another 30 trials with this coherence with the same time limit and goal. Based on the data, we concluded that this was an appropriate difficulty for the task. Data from the third and fourth pilot studies are shown in Table 1, below. We then fitted the data from the final pilot to the LBA. The LBA parameters used for simulating the opponent in the actual experiment are outlined below in Table 2. Each parameter’s mean and standard deviation had its own mean and standard deviation, which were used to randomly sample parameter values for each competitive episode.

**Table 1**

*Average response time, accuracy, and goal achievement rate for the third and fourth pilots.*

|  | Pilot 3 | Pilot 4 |
| --- | --- | --- |
| % of Goals Achieved | 19.4% | 46.3% |
| Average Accuracy | 60.2% | 73.9% |
| Average Response Time | 1.206 | 1.419s |

**Table 2**

*LBA parameter values for the computer opponent*

| Parameter | Mean | SD | Truncated |
| --- | --- | --- | --- |
| B Mean | 0.08 | 0.05 | Yes |
| B SD | 1.02 | 0.26 | Yes |
| A Mean | 1.06 | 0.38 | Yes |
| A SD | 1.86 | 0.43 | Yes |
| V True Mean | 1.72 | 0.22 | No |
| V True SD | 0.66 | 0.23 | Yes |
| V False Mean | 0.78 | 0.19 | No |
| V False SD | 0.57 | 0.18 | Yes |
| Tau Mean | 0.13 | 0.02 | Yes |
| Tau SD | 0.06 | 0.05 | Yes |
| S | 1 | - | - |

**Statistical Analyses**

We examined the effects of time remaining and score difference on response time and accuracy by conducting Bayesian mixed effects models using Bürkner’s (2017) brms package for R. These models included the participant as a random effect. One model examined the effects on accuracy (1 = correct, 0 = incorrect) using a logit link function, and the other model examined the effects on response time in milliseconds using a log link function.

The brms R package estimates parameters using the MCMC routine implemented by the programming language Stan (Carpenter et al., 2017). Weakly informative priors were set for these analyses. For all the accuracy model priors, a Student’s *t*-distribution was used where df = 3, location = 0, and scale = 10. The response time model used the same priors for the fixed effects, whereas the intercept and standard deviation parameters used a *t*-distribution where df = 3, location = 0, and scale = 500. Random effects had a lower bound of 0 and the fixed effects were not truncated. Each model ran four chains with 4000 samples each, where the first 2000 samples for each chain being discarded as warmup. Using the remaining samples, each analysis was based on 8000 samples. Visual inspection of the chains showed excellent mixing, with all R-hat statistics being below 1.02 (except the intercept of the response time model, which was 1.13). The results from the accuracy analysis are presented in Table 3, below, while the outputted results from the response time analysis are presented in Table 4, below.

**Table 3**

*Results of the Bayesian Mixed Modelling Analysis of Accuracy.*

|  | Estimate | SE | Lower CI | Upper CI | Bulk ESS | Tail ESS | Rhat | BF |
| --- | --- | --- | --- | --- | --- | --- | --- | --- |
| Intercept | 1.24 | 0.06 | 1.13 | 1.35 | 452 | 1018 | 1.01 | - |
| Standardized Score Difference | 0.09 | 0.01 | 0.06 | 0.12 | 4540 | 4829 | 1.00 | 120.01 |
| Standardized Score Difference-Squared | 0.002 | 0.005 | -0.01 | 0.01 | 6435 | 558 | 1.00 | - |
| Standardized Time Remaining | 0.01 | 0.01 | -0.02 | 0.03 | 5033 | 5386 | 1.00 | 0.001 |
| Standardized Time Remaining-Squared | -0.0009 | 0.01 | -0.02 | 0.02 | 5213 | 5626 | 1.00 | - |
| Standardized Interaction | 0.02 | 0.01 | -0.01 | 0.04 | 6065 | 5807 | 1.00 | 0.002 |
| Intercept SD | 0.43 | 0.04 | 0.36 | 0.52 | 793 | 1000 | 1.00 | - |

SE represents the standard error. Lower CI and Upper CI represent the lower and upper bounds on the 95% credible interval. Bulk and Tail ESS represent the effective sample size, which measures the sampling efficiency in the bulk and tails of the distribution. Rhat represents the R-hat convergence diagnostic, which is a comparison of the between- and within-chain estimate for the model parameters. BF represents the Bayes factor.

**Table 4**

*Results of the Bayesian Mixed Modelling Analysis of Response Time (ms)*

|  | Estimate | SE | Lower CI | Upper CI | Bulk ESS | Tail ESS | Rhat | BF |
| --- | --- | --- | --- | --- | --- | --- | --- | --- |
| Intercept | 6.90 | 0.03 | 6.83 | 6.97 | 24 | 56 | 1.13 | - |
| Standardized Score Difference | -0.05 | 0.003 | -0.055 | -0.045 | 2722 | 4330 | 1.00 | 21.69 |
| Standardized Score Difference-Squared | 0.002 | 0.0008 | 0.0005 | 0.004 | 11892 | 6389 | 1.00 | - |
| Standardized Time Remaining | 0.05 | 0.002 | 0.04 | 0.05 | 2765 | 4160 | 1.00 | 15.93 |
| Standardized Time Remaining-Squared | -0.03 | 0.002 | -0.04 | -0.03 | 3112 | 4231 | 1.00 | - |
| Standardized Interaction | -0.03 | 0.002 | -0.04 | -0.03 | 3087 | 4542 | 1.00 | 23.56 |
| Intercept SD | 0.27 | 0.02 | 0.23 | 0.32 | 160 | 315 | 1.02 | - |

SE represents the standard error. Lower CI and Upper CI represent the lower and upper bounds on the 95% credible interval. Bulk and Tail ESS represent the effective sample size, which measures the sampling efficiency in the bulk and tails of the distribution. Rhat represents the R-hat convergence diagnostic, which is a comparison of the between- and within-chain estimate for the model parameters. BF represents the Bayes factor.

**Computational Modelling**

The Linear Ballistic Accumulator (LBA) model holds the assumption that evidence for either response alternative accumulates in separate accumulators independently of the other. In this experiment, there were two possible responses, left or right, giving two evidence accumulators. The starting evidence for either alternative response for each decision trial is taken from a uniform distribution [0, *A*]. From this starting point, the evidence accumulates linearly. Each accumulator has a rate of evidence accumulation (the drift rate), which is drawn from a normal distribution with mean *v* and standard deviation *sd*. Evidence is accumulated until enough evidence for one response breaches the threshold (*b*) for a decision to be made, at which point, the response is made. In line with common practice (e.g., Brown and Heathcote, 2008), we express threshold as the difference between the raw threshold and the maximum starting evidence (*B*, where *B* = *b* – *A*). This allows for threshold to be measured purely, without contamination by individual differences in starting evidence. In addition, the LBA also includes a parameter for non-decision time (*t_0_*), which captures the portion of response time that is attributed to other processes besides the decision-making process. These include encoding the stimulus and executing the response manually.

In this experiment, a version of the LBA was used where the mean rates of evidence accumulation for the correct decision varied as a function of time remaining and score difference. It also assumed that threshold (*B*) varied as a function of time remaining and score difference. The starting point variability (*A*), non-decision time (*t_0_*), and the mean rate of evidence accumulation for incorrect responses were constrained across all episodes to be equal. The standard deviation of the drift rate (*sv*) was fixed to one for all episodes and accumulators.

The parameters were estimated using a hierarchical Bayesian framework that assumed that parameters varied across individuals and were drawn from shared population distributions (see Table 5). These population distributions for each parameter have two hyperparameters, being location (μ) and scale (σ). The hyperparameters were chosen to be weakly informative. The parameters at the participant level were modeled using either normal or truncated normal distributions. Both the *A* and *B* parameters were set with a lower bound of 0 and no upper bound. The *t_0_* parameter was constrained between 0.1 and 1, while the *v* parameter was not truncated at all. The priors used were based on methods used by Gronau, Heathcote, and Matzke (2019).

**Table 5**

*Priors for the Population Distributions.*

| Population Distribution | Model Parameter | Distribution Family | Mean | SD | Lower | Upper |
| --- | --- | --- | --- | --- | --- | --- |
| Location | *A* | Truncated Normal | 1 | 1 | 0 | None |
|  | *B intercept* | Truncated Normal | 0.4 | 0.4 | 0 | None |
|  | *B on deadline* | Standard Normal | 0 | 1 | None | None |
|  | *B on score difference* | Standard Normal | 0 | 1 | None | None |
|  | *B on interaction* | Standard Normal | 0 | 1 | None | None |
|  | *v true intercept* | Normal | 3 | 3 | 0 | None |
|  | *v true on deadline* | Standard Normal | 0 | 1 | None | None |
|  | *v true on score difference* | Standard Normal | 0 | 1 | None | None |
|  | *v true on interaction* | Standard Normal | 0 | 1 | None | None |
|  | *v false* | Normal | 1 | 1 | None | None |
|  | *t_0_* | Truncated Normal | 0.3 | 0.3 | 0.1 | 1 |
| Scale | *A* | Truncated Normal | 1 | 1 | 0 | None |
|  | *B* | Truncated Normal | 0.4 | 0.4 | 0 | None |
|  | *v true* | Truncated Normal | 3 | 3 | 0 | None |
|  | *v false* | Truncated Normal | 1 | 1 | 0 | None |
|  | *t_0_* | Truncated Normal | 0.3 | 0.3 | 0 | None |

The posterior distributions were estimated using the Hamiltonian Monte Carlo algorithm implemented by Stan (Annis, Miller, & Palmeri, 2016; Carpenter et al., 2017). Four chains were run, each with 2000 iterations. 1000 of these iterations were run for sample burn in, and were discarded, leaving 1000 iterations. Visual inspection of the chains showed excellent mixing and stationarity.

Figure 3 shows the proportion of correct responses for both the observed and predicted data separated over deadline conditions overall and score distance at the time of each decision. Figure 4 shows the same, except for response times. In order to calculate the observed values for both figures, the relevant value (proportion of correct responses or response time) were averaged for each participant, and these were then averaged across participants. The predicted values were calculated through the same procedure for each sample, allowing a full posterior distribution to be obtained for each value. The model provides relatively close fit to the data, capturing and allowing enough complexity to capture the patterns of the data while still maintaining simplicity. Overall, the model provided a good visual account of the data, particularly between the 10th and 50th quantiles, though there is some misfit at the 90th quantile. With longer deadlines, participants took longer to make decisions, whereas shorter decisions were made with shorter deadlines, fitting the model better. This may be evidence of a non-linear effect of deadline on response time, but we did not introduce that kind of complexity into the model for simplicity’s sake.

**Figure 3**

*Mean prorportions of correct responses for the real (observed) data and the simulated (predicted) data. The error bars for the predicted data represent the 95% credible interval. The left graph shows the results for the “long conditions” (time limit of 20 or 40 seconds) and the right graph shows the results for the “short conditions” (time limit of 5 or 10 seconds). On each graph, the left point shows the results for “close” decisions (participant is within two points of their opponent in either direction), the middle point shows “losing” decisions (participant is three or more point behind their opponent), and the right point shows the results for “winning” decisions (participant is three or more points ahead of their opponent).*


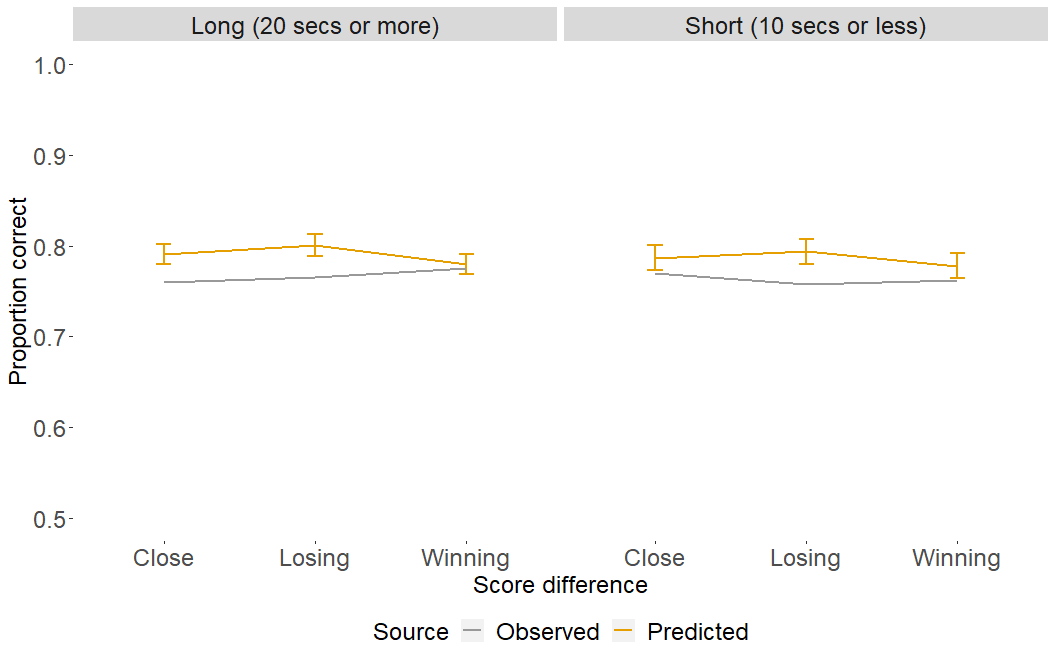


**Figure 4**

*The mean 10^th^, 30^th^, 50^th^, 70^th^, and 90^th^ quantiles for the response time distributions that were observed and predicted by the model. The left graphs show competitions that lasted 20 or 40 seconds, and the right graphs show competitions that lasted 5 or 10 seconds. The top graphs show decisions that were “close” (partipants were within two points ahead or behind their opponents). The middle graphs show decisions where the participant was “losing” (participants were three or more points behind their opponents). The bottom graphs show decisions where the participant was “winning” (participants were three or more points agead of their opponents). The top lines show correct responses and the bottom lines show incorrect responses. The error bars for the predicted data represent the 95% credible interval.*


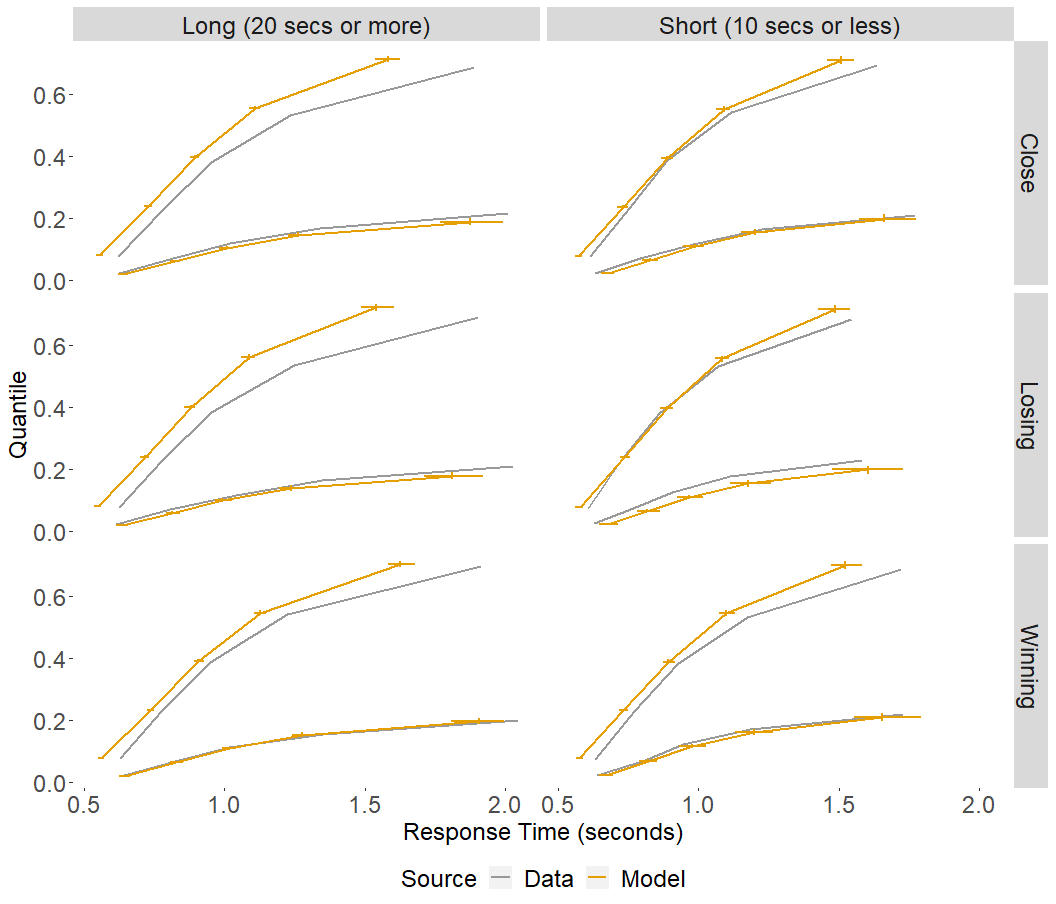


Parameter recovery analysis was conducted to determine whether the estimated parameters were recoverable. Simulated data, with the same number of trials as the real data, were generated using each participants’ mean value in each condition for each parameter. This allowed for comparison between the data generating parameters and the estimated parameters. Figures 5 and 6, below, show the data-generating and recovered parameters graphed against each other. Figure 5 shows the threshold parameters for each condition and Figure 6 shows the difference between drift rate parameters for each condition. Based on this analysis, the recovery of the parameters was mostly good, meaning the parameters are well estimated given the sample size and design. The effect of deadline on drift rates parameter did not recover well and should be interpreted accordingly. This poor recovery was matched with weaker evidence of an effect. Figure 7 shows the parameter recoveries for the remaining parameters.

**Figure 5**

*Actual and recovered values for the mean threshold parameter for deadline, score difference, and the interaction.*


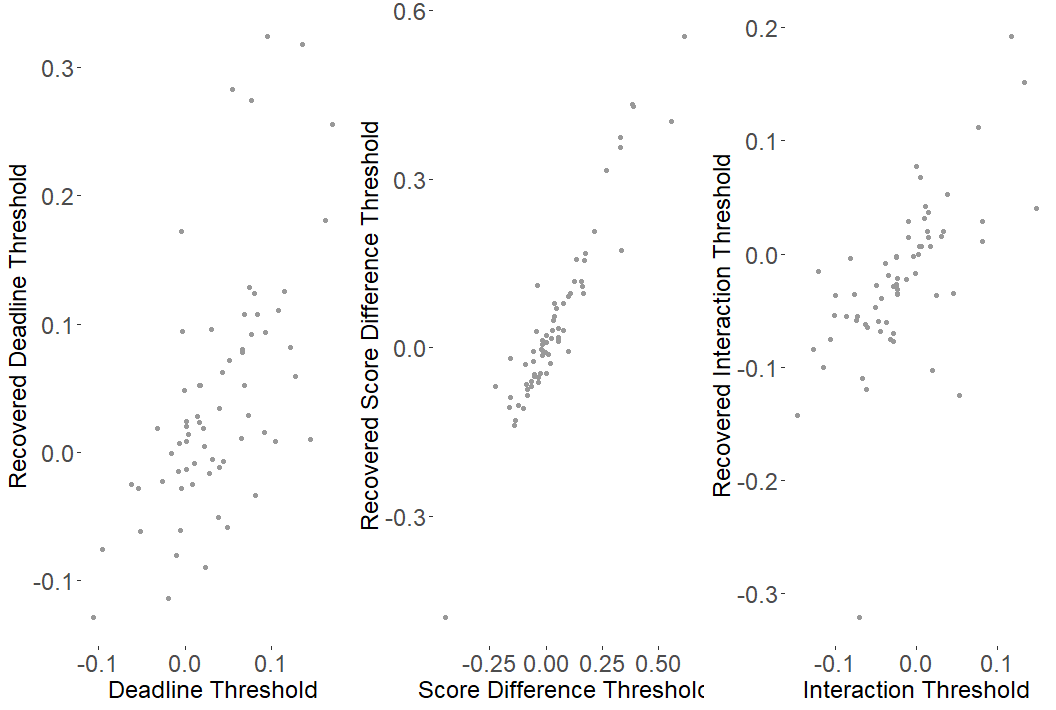


**Figure 6**

*Actual and recovered values for the mean drift rate parameter for the correct decision for deadline, score difference, and the interaction.*


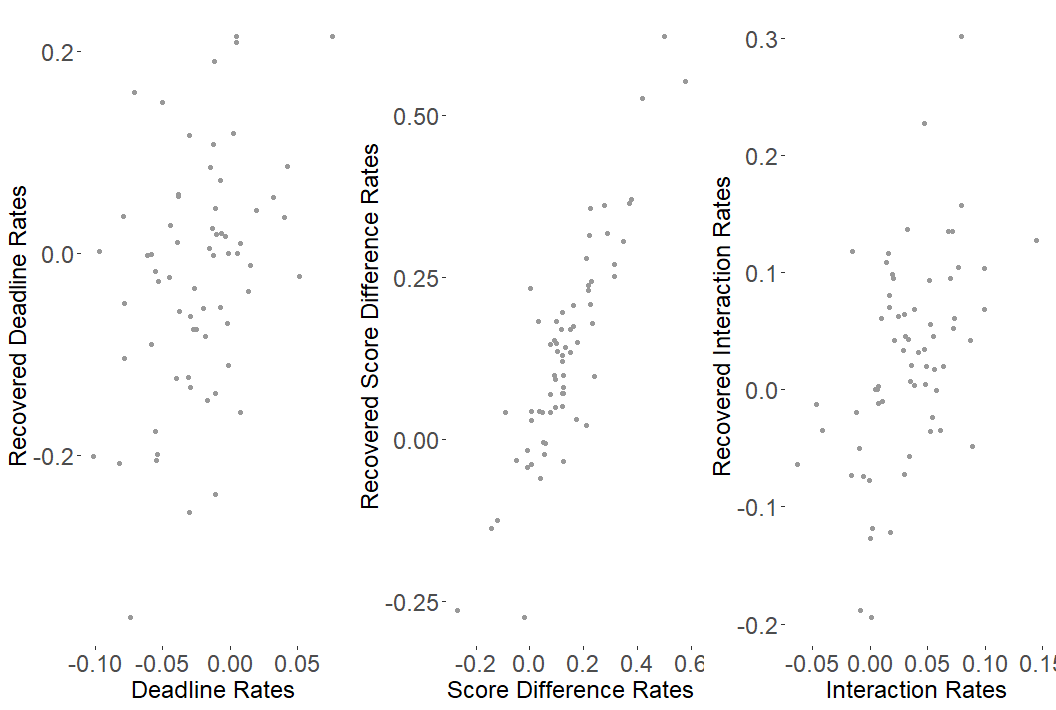


**Figure 7**

*Actual and recovered values for the mean incorrect drift rate, mean starting evidence, and mean non-decision time.*


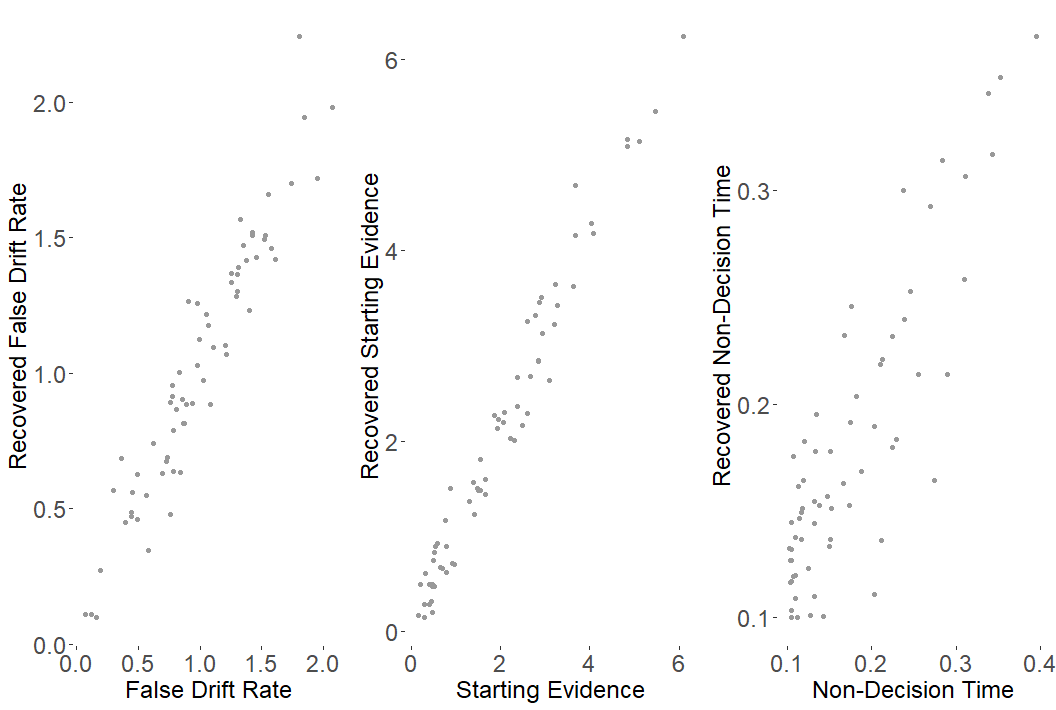

Supplement: Supplementary file 1 — Supplementary file1 (DOCX 281 KB) [file 13423_2024_2523_MOESM1_ESM.docx]
